# An extraordinary fossil captures the struggle for existence during the Mesozoic

**DOI:** 10.1038/s41598-023-37545-8

**Published:** 2023-07-18

**Authors:** Gang Han, Jordan C. Mallon, Aaron J. Lussier, Xiao-Chun Wu, Robert Mitchell, Ling-Ji Li

**Affiliations:** 1 Hainan Vocational University of Science and Technology, Haikou, Hainan China; 2 Hainan Tropical Ocean University, Sanya, Hainan China; 3 Beaty Centre for Species Discovery and Palaeobiology Section, Canadian Museum of Nature, Ottawa, Ontario Canada; 4 Department of Earth Sciences, Carleton University, Ottawa, Ontario Canada; 5 Beaty Centre for Species Discovery and Mineralogy Section, Canadian Museum of Nature, Ottawa, Ontario Canada; 6 Department of Geography, University of Calgary, Calgary, Alberta Canada; 7 Weihai Ziguang Shi Yan School, Weihai, Shandong China

**Keywords:** Palaeontology, Palaeoecology

## Abstract

Dinosaurs and mammals have coexisted for the last ~ 230 million years. Both groups arose during the Late Triassic and diversified throughout the Mesozoic and into the Cenozoic (the latter in the form of birds). Although they undoubtedly interacted in many ways, direct fossil evidence for their interaction is rare. Here we report a new fossil find from the Lujiatun Member of the Lower Cretaceous Yixian Formation of China, showing a gobiconodontid mammal and psittacosaurid dinosaur locked in mortal combat. We entertain various hypothesized explanations for this association, but the balance of the evidence suggests that it represents a predation attempt on the part of the smaller mammal, suddenly interrupted by, and preserved within, a lahar-type volcanic debris flow. Mesozoic mammals are usually depicted as having lived in the shadows of their larger dinosaurian contemporaries, but this new fossil convincingly demonstrates that mammals could pose a threat even to near fully-grown dinosaurs. The Yixian Formation—and the Chinese fossil Jehol Biota more broadly—have played a particularly important role in revealing the diversity of small-bodied dinosaurs and other fauna. We anticipate that the volcanically derived obrution deposits specific to the Lujiatun Member will likewise continue to yield evidence for biotic interactions otherwise unknown from the rest of the fossil record.

## Introduction

For nearly 230 million years, mammals and dinosaurs have been among the most successful vertebrate groups on Earth. Each group originated in the Late Triassic and has since diversified into the myriad of forms known today (dinosaurs in the form of birds, following the end-Cretaceous mass extinction). Mesozoic mammals are commonly depicted as having lived in the shadows of their larger dinosaurian contemporaries, and dinosaur gut contents containing the remains of small mammals bear this out^1–3^. It was not until after the end-Cretaceous extinction of the non-avian dinosaurs that mammals grew to large body sizes and regularly preyed on the available avifauna.

Dinosaur-mammal antagonism during the Mesozoic was not strictly unilateral, however. Fossil gut contents of the Chinese gobiconodontid mammal *Repenomamus robustus* contain the remains of a much smaller, perinatal *Psittacosaurus* sp.^4^, an early ceratopsian dinosaur. Here we report on a yet more exceptional discovery preserving these two genera locked in mortal combat—the first such fossil of its kind. The new fossil (Weihai Ziguang Shi Yan School Museum WZSSM] specimen VF000011) was discovered on May 16, 2012 west of Lujiatun Village in Liaoning Province, near coordinates N41°36′24″, E120°54′40″ (Supplementary Fig. S1). It was acquired by the first author and donated to the WZSSM in 2020. The fossil originates from the Lujiatun Member of the Yixian Formation, the latter being 212 m thick locally. The Lujiatun Member is famous for its abundance of vertebrate fossils, especially the fossils of *Psittacosaurus lujiatunensis*, whose uncorrected relative abundance reaches nearly 90%, locally^5^. The sediments there are volcanically derived, and although depositional age estimates have varied^6,7^, the latest U–Pb dating of tuffaceous zircons indicates an age between 125.755 ± 0.061 and 125.684 ± 0.060 Ma^8^.

## Results and discussion

### Analysis of host rock

To better constrain depositional history, a single sample (~ 5 × 5 × 2 cm) of moderately-to-well consolidated sedimentary material, found next to the skeletons, was acquired for investigation. Thin section analysis showed the rock to be a matrix-supported, medium-coarse brecciated tuff, following the classification scheme of White and Houghton^9^.

Framework components are either lithic fragments (i.e. being multi-phase and retaining initial texture) or mineral fragments (i.e. being > 90% a single phase). Sizes range from 0.02 to 5.96 mm with a mean of 0.46 mm (lithic fragments), and 0.02–0.43 mm with a mean of 0.11 mm (mineral fragments). Of the 228 mineral fragments characterized, 73% and 16% are either feldspar or quartz, respectively, with less abundant phases including Fe/Ti-oxides (6%), biotite-phlogopite (2%), amphiboles (1%), pyroxenes (1%), and apatite (1%; Supplementary Fig. S2A). Of the 169 lithic clasts characterized, 88%, 8%, and 4% were determined to be of igneous (volcanic), unknown, or sedimentary precursor types, respectively (Supplementary Fig. S2B). Those of volcanic origin are commonly (~ 60%) observed to contain phenocrysts. Selected examples are shown in detail in Supplementary Fig. S3. Matrix material consists predominantly of fine ash-sized smectite (nontronite), quartz, feldspar, and heulandite (Supplementary Fig. S4). Further, thin section mapping shows an average matrix: framework clast ratio of 70:30. In the limited amount of material available for study, neither sedimentary structures (such as grading or stratification) nor bioturbation features are observed.

Previous investigations of the Yixian Formation have suggested sedimentary material to have originated from multiple volcanic events, which caused associated lahars, pyroclastic flows, and ashfalls^10,11,12,13^. Here, the observed diversity in lithic clast characteristics, such as phenocryst phase composition (Supplementary Fig. S2C) and colour (Supplementary Fig. S2D), is consistent with these having originated from multiple (though predominantly volcanic) primary lithologies, consistent with the high degree of physical mixing associated with a pyroclastic origin. High-energy deposition is also evidenced by poor sorting, high angularity, and variable roundness (Supplementary Fig. S5), as well as the common presence of truncated phenocrysts at edges of lithic fragments (Supplementary Fig. S3). Calcite cement is also observed, commonly occurring at the margins of individual lithoclasts as well as in fractures throughout the rock (Supplementary Fig. S3). The abundance of smectite (nontronite) in the matrix material suggests diagenetic alteration of original mafic volcanic materials.

### Assessment of preserved individuals

**Figure 1:**
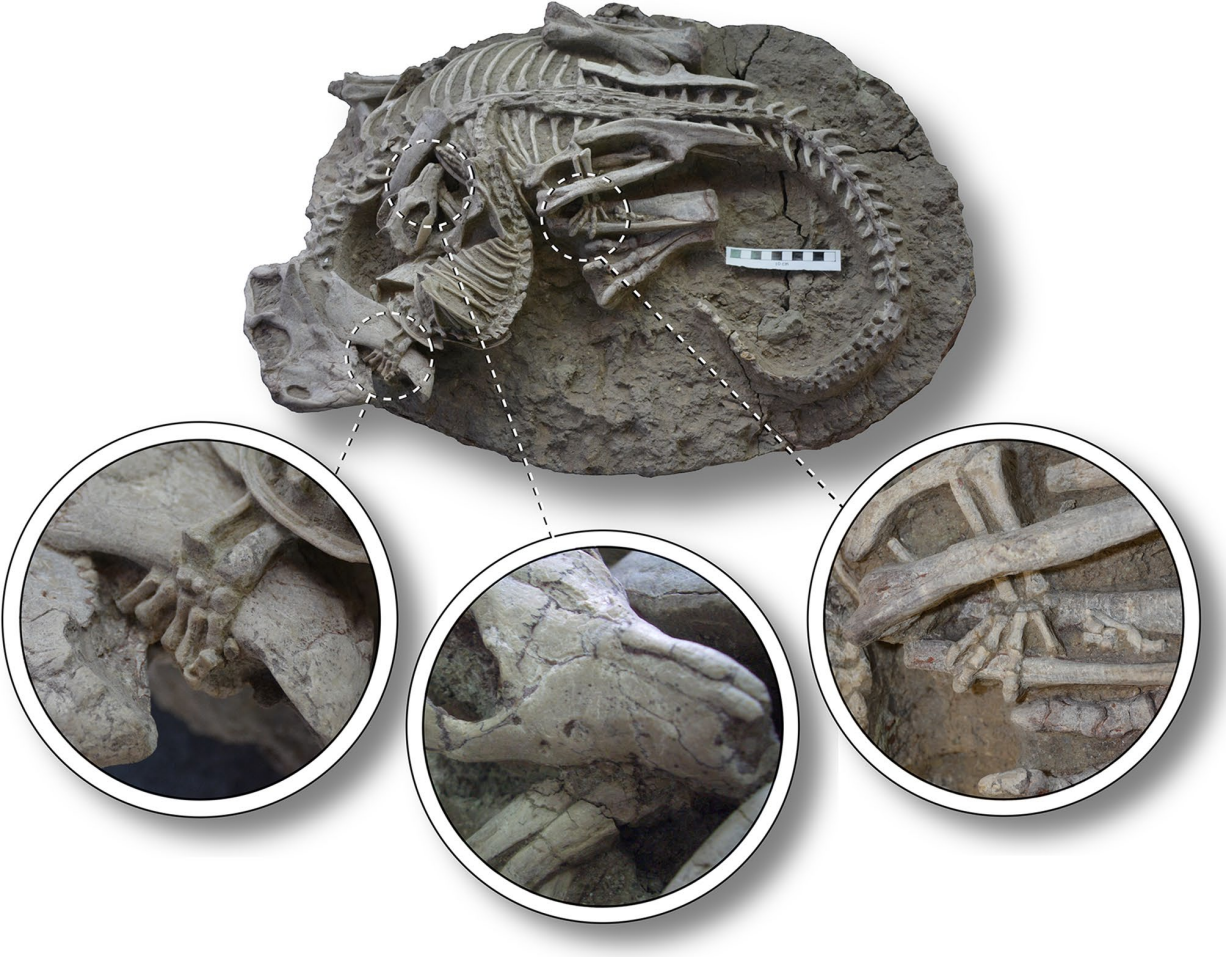
*Psittacosaurus lujiatunensis*-*Repenomamus robustus* pair (WZSSM VF000011) locked in mortal combat. Insets depict (left to right): hand of R. *robustus* wrapped around lower jaw of *P. lujiatunensis*, teeth of *R. robustus* embedded in ribs of *P. lujiatunensis*, hind foot of *R. robustus* wrapped around lower hindlimb of *P. lujiatunensis*. Scale bar equals 10 cm.

The entombed individuals represent the small ceratopsian dinosaur *Psittacosaurus lujiatunensis* entangled with the even smaller gobiconodontid mammal *Repenomamus robustus* (Fig. 1, Supplementary Fig. S6). Skeletal measurements for both are given in Table 1.

**Table 1:** Measurements for *Psittacosaurus lujiatunensis*-*Repenomamus robustus* pair (WZSSM VF000011) in mm.

Measurement	*Psittacosaurus lujiatunensis*	*Repenomamus robustus*
Basal skull length (snout to occipital condyle)	157.5	79.5
Skull length (maximum)	179.7	90.8
Skull width (maximum)	156	51.2
Humerus length	78.6	56.7
Humerus minimum shaft circumference	13.6	11.4
Femur length	137.7	58.8
Femur minimum shaft circumference	17.7	7.3
Trunk length (atlas to posterior sacral vertebra)	564.8	315.2
Tail length (as preserved)	473.9	73.3
Total body length (including skull)	1196.1	467.9

The dinosaur skeleton is complete. The skull preserves all three autapomorphies that diagnose *P. lujiatunensis* (prefrontal width less than 50% that of the nasal, quadratojugal-squamosal contact along the anterior margin of the quadrate shaft, jugal-quadrate contact posteroventral to the laterotemporal fenestra; Supplementary Fig. S7)^14^. The dinosaur is lying prone, with its hindlimbs folded on either side of the body. The neck and tail curl to the dinosaur’s left. Based on femoral circumference scaling^15^ and applied developmental mass extrapolation^16^, we estimate its body mass to have been 10.6 ± 6.0 kg at time of death. We estimate its age to have been at least 6.5 years, and more likely closer to 10 years, based on established femur length-age relationships^17,18^.

The body of the mammal coils to the right and sits atop the left side of the dinosaur. The skeleton is nearly complete; only the distal end of the tail is missing. In life, the mammal would have been several centimeters longer than preserved (Table 1). Although diagnostic dental and mandibular characters are poorly exposed on the individual and so cannot be verified, the comparably small size of the animal, and its weak sagittal and lambdoid crests and zygomatic arches (Supplementary Fig. S8), suggest that it is *R. robustus* and not the larger *R. giganticus*, which is also known from the Lujiatun Member^4^. The mammal’s left hand grips the lower jaw of the dinosaur (which is dislocated and displaced rostrally), and its left hindleg is trapped within the folded left leg of the dinosaur, the hindfoot gripping the dinosaur’s left shin, immediately below the knee (Fig. 1; Supplementary Fig. S9). The mammal died while biting two of the dinosaur’s left anterior dorsal ribs; its mandible plunges downward into the indurated sediment to firmly clasp the bones (Fig. 1; Supplementary Fig. S9). These two ribs appear to be broken, based on their slight misalignment with the remaining ribcage, but the breaks are obscured, and it is not possible to determine with certainty whether the ribs were broken in life or due to taphonomic processes. Developmental mass extrapolation and long bone circumference-body mass scaling relationships provide a mass of 3.43 ± 1.42 kg for the mammal. Cheek tooth eruption and wear are commonly used as indicators of maturity in mammals^19^, but this is not possible for the implicated *R. robustus* individual because the teeth are obscured. The femur of the mammal is 15% shorter than that of the *R. robustus* holotype (Institute of Vertebrate Paleontology and Paleoanthropology [IVPP] specimen V 12549), and the obliteration of the internasal and interfrontal sutures (Supplementary Fig. S8) does not appear as extensive as in the holotype. Nevertheless, the long bone epiphyses of the mammal are fused, indicating that growth was nearing cessation. Probably, this individual was a subadult when it died.

### Assessment of fossil association

The intimate and intertwined nature of the skeletons is remarkable, and suggests that this fossil association is authentic, not forged. Although fossil forgeries have been reported from the Jehol Group of China before, these typically involve the simple juxtaposition of two or more independent fossils^20,21^, and do not replicate the tangled nature of the skeletons documented here. It might be argued that the broken and slightly displaced anterior ribs of the dinosaur indicate tampering, given the otherwise mostly intact nature of the skeletons. However, there has been postdepositional displacement of some other bones, including the lower jaw of the dinosaur (Supplementary Fig. S6B), and the distal manual and pedal phalanges (Fig. 1, Supplementary Fig. S9A,C) and distal tail vertebrae of the mammal (Fig. 1); similar displacement of the ribs is therefore possible, particularly if they had fractured prior to burial. To convince ourselves of the authenticity of the fossil, we prepared and exposed the left dentary of the mammal, which had not yet been revealed at the time of acquisition, and found that it, too, plunges into the matrix to clasp the dinosaur’s ribs (Supplementary Fig. S10).

The association of the two animals also could not have resulted from passive taphonomic processes; the intact nature of the skeletons (except for some of the lighter distal limb and tail elements) indicates that they could not have been transported any appreciable distance prior to deposition. Rather, the animals almost certainly were buried where they died, both events having occurred closely in time, if not simultaneously.

The clutching hands and feet of the mammal, its biting jaws, and its position atop the dinosaur indicate that the mammal was clearly the aggressor in the preserved interaction, in agreement with the inferred carnivorous lifestyle of *Repenomamus robustus*^4^ (*Psittacosaurus lujiatunensis* was almost certainly herbivorous^14^). Nevertheless, the nature of the interaction is not immediately obvious. It is possible that the mammal was scavenging the carcass of the dinosaur when the two became buried. This proposed scenario would account for the large size of the dinosaur relative to the mammal (terrestrial predators usually favour prey that are not much larger than themselves, particularly when hunting alone^22,23^), and the fact that the mammal was biting the ribs of the dinosaur when it died, which would otherwise have been difficult to access (but not impossible—see below) on a living prey item. However, while plausible, we cite three lines of evidence that challenge this hypothesis. First, the bones of the dinosaur are otherwise devoid of tooth marks, which are commonly left by carnivorous mammals while scavenging^24^. Second, it seems unlikely that the two animals would have become so entangled, were the dinosaur dead prior to the arrival of the mammal. Third, the scavenging scenario does not predict the position of the mammal atop the dinosaur, since the mammal could presumably just as easily have eaten the dinosaur from ground level.

**Figure 2:**
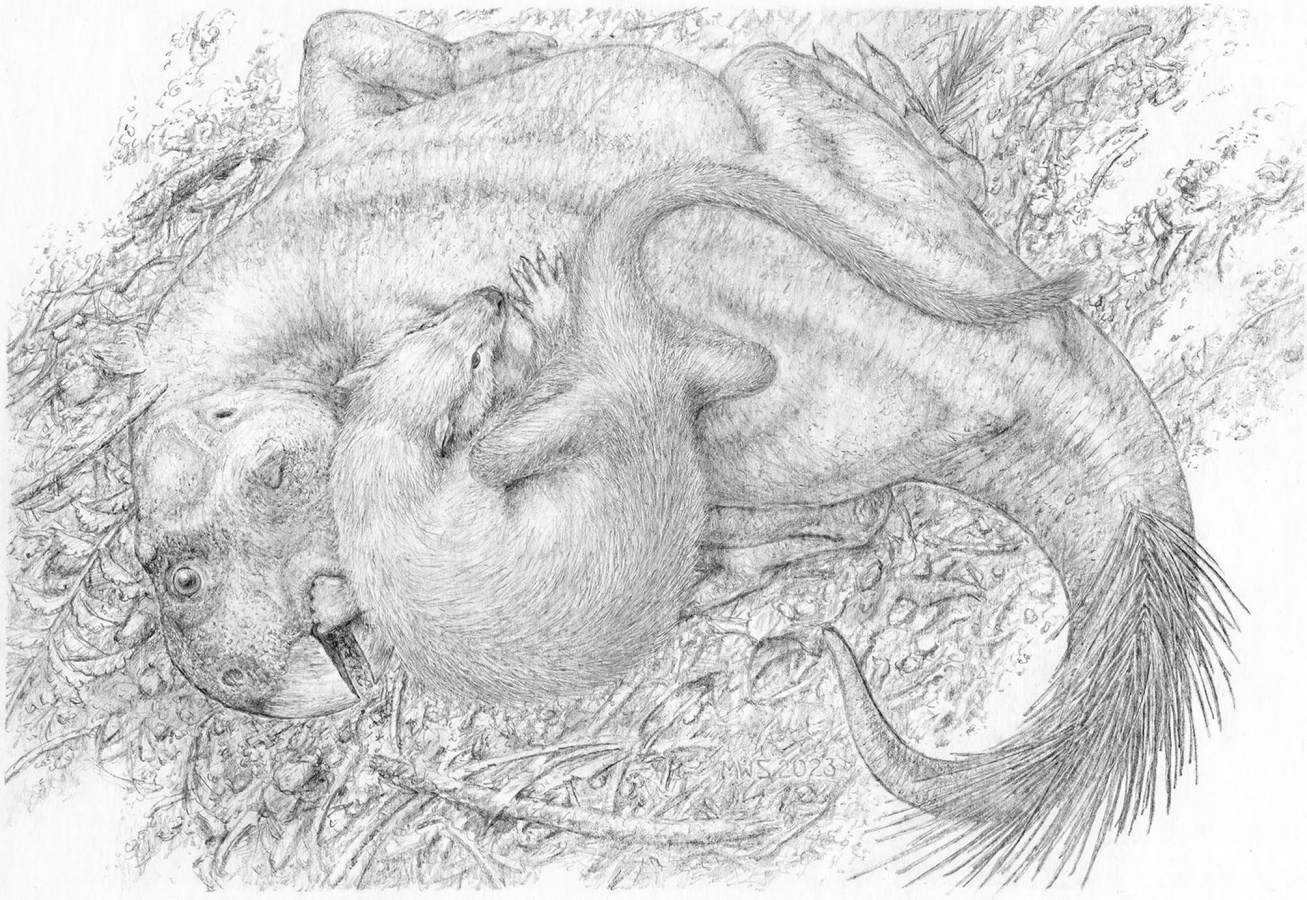
Life restoration showing *Repenomamus robustus* grappling with *Psittacosaurus lujiatunensis*. Artwork by Michael Skrepnick. Reproduced with permission.

**Figure 3:**
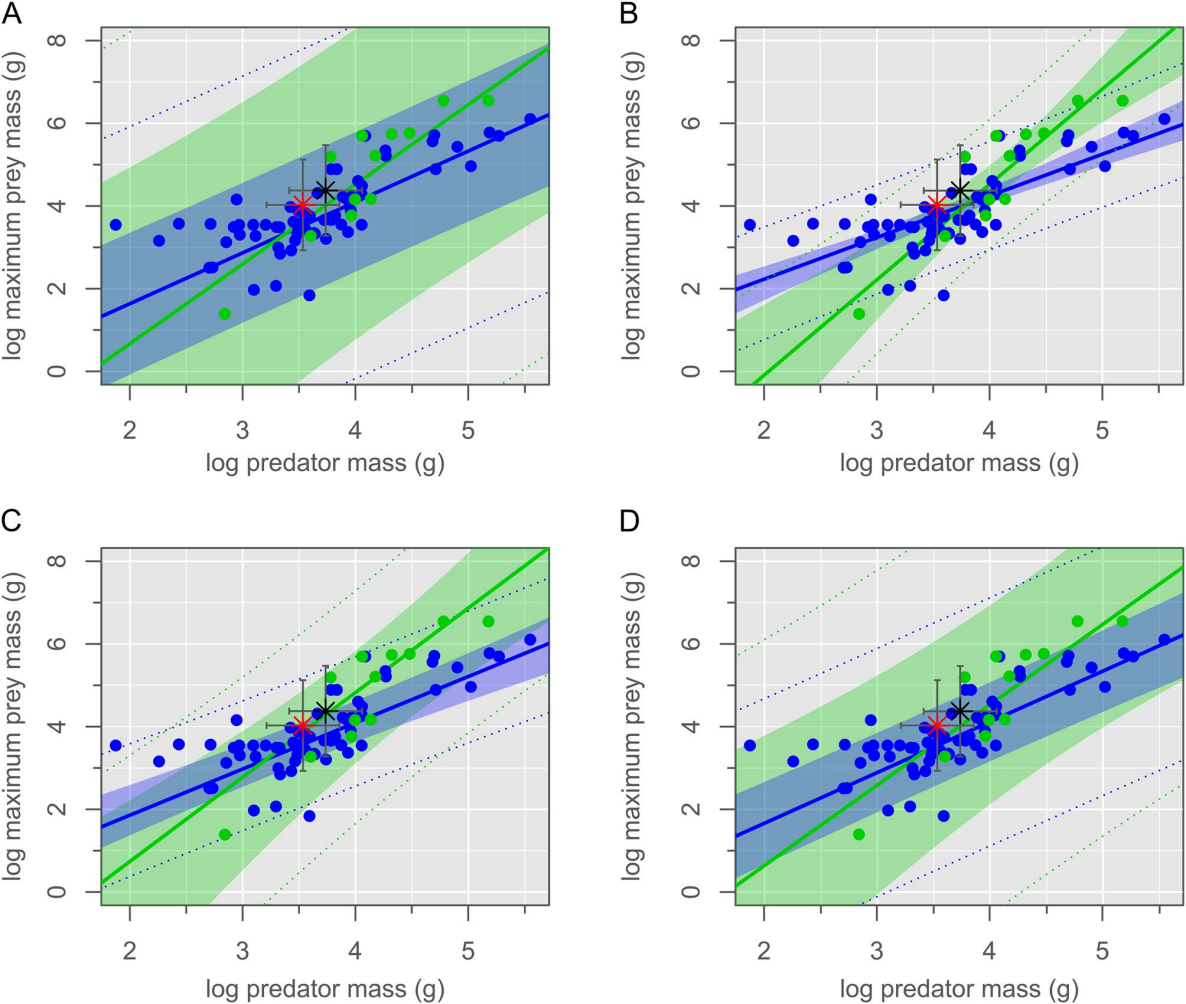
Phylogenetic generalized least squares models. (**A**) Brownian motion, (**B**) Ornstein–Uhlenbeck, (**C**) Pagel’s λ, (**D**) ACDC. Linear models show the relationship between predator body mass and maximum prey body mass for solitary (blue) and pack (green) hunters. Shaded areas represent the 95% confidence intervals; dotted lines represent 95% prediction intervals. The association between the *Repenomamus robustus* and *Psittacosaurus lujiatunensis* documented here (red star), and the predicted association of somatically mature examples of these species (black star), are well within the 95% prediction intervals for each model and hunting style.

We propose instead that the two animals were buried in an act of predation on the part of the mammal, only for both to have been entombed by a sudden lahar-type volcanic debris flow (Fig. 2). This hypothesis would explain the entwined nature of the skeletons, wherein the left hindfoot of the mammal became trapped within the folded left leg of the dinosaur when it collapsed to the ground. It would also account for the lack of tooth marks and other indications of scavenging on the dinosaur’s skeleton, and for the mammal’s position atop the dinosaur, as though to subdue its weakened prey. To address the question of whether the dinosaur was too large to have been reasonably preyed upon by the mammal, we examined the relationship between predator body mass and prey maximum body mass among terrestrial carnivorans using phylogenetic generalized least squares (PGLS) regression. We modeled several evolutionary scenarios, of which a Pagel’s lambda model best fits the data (Fig. 3, Supplementary Table S2). Our fossil association falls well within the 95% prediction intervals for both solitary and pack hunters. We therefore cannot reject the hypothesis that this association preserves a doomed predation event on the part of the mammal, despite its smaller size. By analogy, although wolverines (*Gulo gulo*) are typically opportunistic feeders of large prey, lone individuals are also known to occasionally hunt animals many times their own size, including moose (*Alces alces*), caribou/reindeer (*Rangifer tarandus*), and domestic sheep (*Ovis* spp.)^25,26^. Least weasels (*Mustela nivalis*) have similarly been reported as occasionally attacking much larger capercaillie (*Tetrao* spp.), hazelhen (*Tetrastes bonasia*), and hare (*Lepus* spp.)^27,28^.

It may seem unlikely that the mammal was biting the exposed ribs of the dinosaur if it were not already long deceased; however, feeding on live prey happens commonly in carnivorous mammals, including African wild dogs (*Lycaon pictus*), spotted hyenas (*Crocuta crocuta*), and jackals (*Canis mesomelas* and *C. aureus*)^29,30^. In fact, after an initial struggle, the prey may ultimately give up on self-defence, opting instead to passively lay down in a state of exhaustion and deep shock^29^. This depiction is not very unlike the position assumed by the dinosaur described here. Kleptoparasitism by large predators on the open African savannah can significantly alter the hunting and feeding habits of smaller species^31,32^, and the eating of still-living prey by African wild dogs is conceivably one such adaptation. The larger carnivorous theropods of the Early Cretaceous Lujiatun ecosystem might have posed an equal threat to *Repenomamus* spp., motivating a similarly rapacious feeding behaviour in the mammals.

**Figure 4:**
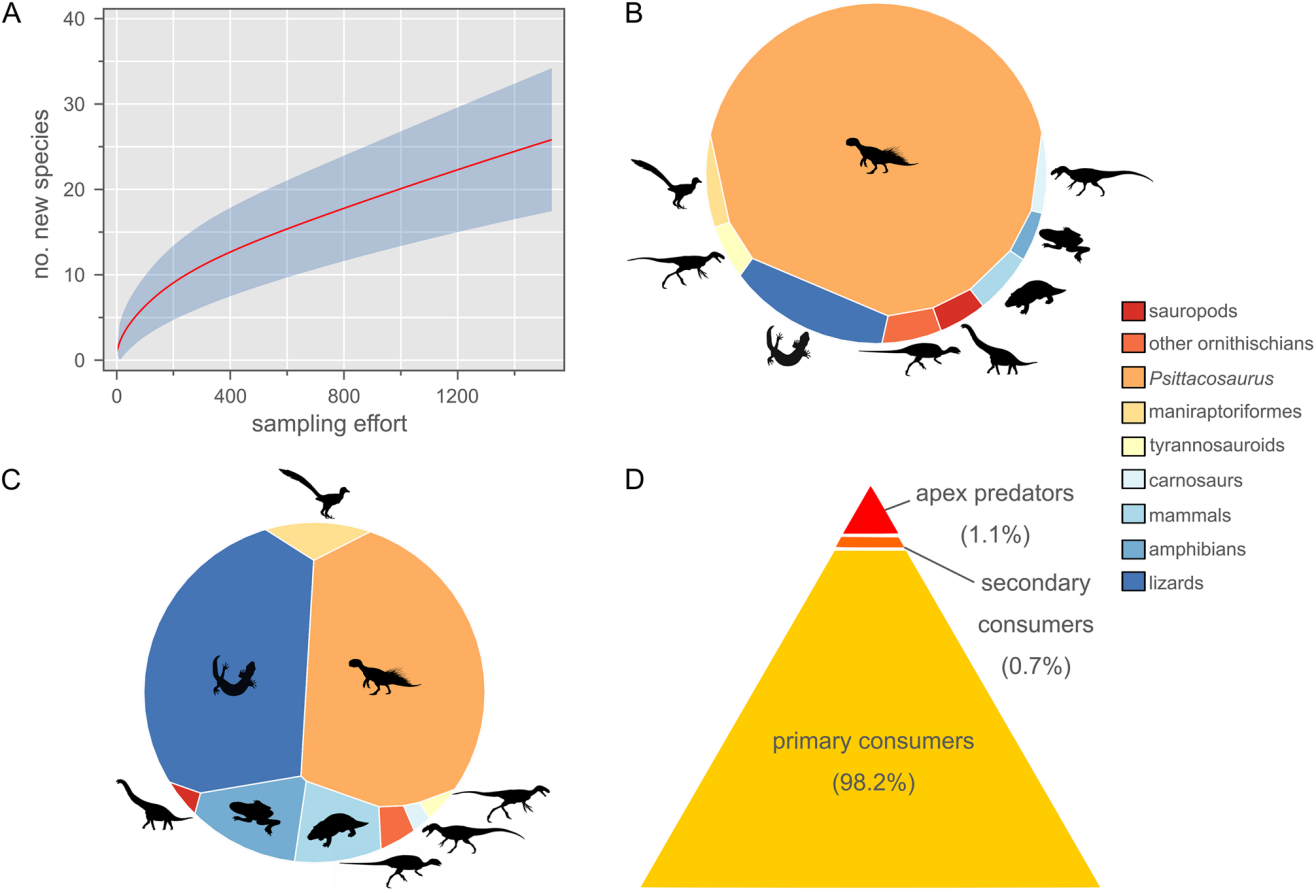
Species diversity statistics for the Lujiatun Member of the Lower Cretaceous Yixian Formation of China. (**A**) Rarefaction curve showing undersampled richness of Lujiatun Member. (**B**) Voronoi diagram showing uncorrected relative abundance data. (**C**) Voronoi diagram showing relative abundance corrected for taphonomic size bias. (**D**) Trophic biomass pyramid corrected for taphonomic factors. *Psittacosaurus lujiatunensis* makes up 85.9% of all primary consumer biomass in the restored terrestrial vertebrate fauna. Silhouette credits (from phylopic.org): DiBgd (sauropod); T. Dixon (maniraptoriform); S. Hartman (carnosaur, tyrannosauroid); J. Headden (*Psittacosaurus*); N. Mongiardino Koch (lizard); V. Sinkkonen (ornithischian); E. Willoughby (amphibian); M. Zica (mammal).

Could an adult *P. lujiatunensis* have eventually outgrown the prey size threshold of *R. robustus*, and thereby avoided further predation from the latter species? We examined this question in the same way as above, plotting the adult body masses of these two species on our regression estimates (Fig. 3). Again, an adult *P. lujiatunensis* (body mass ≈ 23.5 kg) plots well within the expected maximum prey size threshold of an adult *R. robustus* (body mass ≈ 5.54 kg). It is therefore plausible that *P. lujiatunensis* remained vulnerable to predation from *R. robustus* throughout its lifetime. Threats from the still larger *Repenomamus giganticus*, the tyrannosauroid *Dilong paradoxus*, and an as-yet undescribed carnosaur ensured that *P. lujiatunensis* were ever-vigilant during ‘Lujiatun time’. Despite undersampling of the otherwise fossiliferous Lujiatun Member (Fig. 4A), it is clear that *P. lujiatunensis* was an abundant prey item on the Early Cretaceous landscape, considering raw and taphonomically-corrected count data and estimated standing crop biomass (Fig. 4B–D).

## Conclusions

Dinosaurs and mammals lived alongside one another throughout most of the Mesozoic, but direct fossil evidence for their interaction is rare^1–3^^,^^33,33,35^. An extraordinary new fossil (WZSSM VF000011) from the Lujiatun Member of the Lower Cretaceous Yixian Formation in China preserves a mammal (*Repenomamus robustus*) and dinosaur (*Psittacosaurus lujiatunensis*) in close association. Their entwined skeletons suggest that the fossil is not a forgery, and the completeness of the skeletons indicates that they were not transported prior to burial. The lack of bite marks on the dinosaur skeleton, the position of the mammal atop the dinosaur, and the grasping and biting actions of the mammal collectively signal that the mammal was preying on the weakened dinosaur when the two were suddenly entombed by a volcanic debris flow. The dinosaur is 3 × larger than the mammal by estimated body mass, but the fossil association falls well within the 95% prediction intervals for a linear model of maximum prey body mass vs. predator body mass among terrestrial carnivorans, for both solitary and pack hunters. The new fossil thus challenges the common assumption that Mesozoic mammals were merely fodder for the ruling dinosaurs.

Obrution deposits like the one reported here provide unique information about fossil behaviour that is otherwise not preserved in time-averaged settings. The Lujiatun Member of the Yixian Formation, previously referred to as the “Chinese Pompeii”^13^, stands out for its preservation of multiple such deposits containing spectacular, three-dimensional vertebrate body fossils. The potential to preserve similar ‘snapshots’ of dinosaur behaviour is equaled only by the ichnological record^36^ and by few other skeletal deposits worldwide (e.g. ref.^37^). The Chinese fossil Jehol Biota, more broadly, is of further importance because it does not suffer from the same taphonomic bias against the preservation of small vertebrate fossils commonly seen in high-energy fluvial deposits elsewhere, which can obscure palaeoecological reconstructions^38,39^. These key factors single out the Lujiatun Member as uniquely insightful regarding dinosaur-dominated ecosystems, and we anticipate that future fossil discoveries—combined with the diligent collection of associated locality data—will ensure the success of the Lujiatun Member as a sort of ‘natural laboratory’ for the study of life during the Cretaceous.

## Methods

### Analysis of sedimentary material

From a single hand sample (5 × 5 × 2 cm, approx.), a suite of four polished thin sections (labelled L-TS-1 through L-TS-4) were prepared at the petrographic laboratories of the University of Ottawa, Department of Earth and Environmental Sciences. To preserve the integrity of the moderately-to-well consolidated bulk material, sections were polished to thicknesses greater than the standard 30 μm used in petrographic sections. Careful examination of the sections by petrographic and binocular microscope showed each to be consistently representative of the bulk material in the hand sample. Thin section L-TS-2 was selected for the more detailed analyses.

For L-TS-2, the complete section was imaged with a JEOL 8230 electron microprobe operating in backscatter imaging mode with a beam diameter of 10 mm and an accelerating voltage of 20 keV, and the resulting images were integrated using the MIST software package^40^ to create a continuous image.

Individual clasts were characterized using features embedded within the Adobe Illustrator 2022 software package. A total of 383 individual fragments, distributed randomly throughout the thin section, were visually selected for investigation. For each clast, the following characteristics are considered: size, composition, shape (roundness and sphericity), colour, and presence of alteration features. Roundness, sphericity, and clast size were calculated as follows. The equation of Wadell^41^ *R* = ${\sum }_{i=1}^{n}({r}_{i} / {r}_{max})/ n$ is used to quantify fragment roundness (in 2-dimensions). Here, *r*_*i*_ is the radius of curvature of the *n*^*th*^ fragment corner, and *r*_*max*_ is the radius an inscribed circle of maximal diameter. Sphericity was calculated using the method of Krumbein and Sloss^42^, ψ = *D*_*min*_/*D*_*max*_, where *D*_*min*_ and *D*_*maz*_ are defined as the minimal and maximal cross-sectional dimensions, respectively. The size of each clast is taken as the average cross-sectional dimension, *S* = (*D*_*min*_ + *D*_*max*_)/2. The colour of each lithic clast is determined by optical examination in white, non-polarized light using a binocular microscope. Colours are grouped by dominant hue. Subset sizes were as follows: roundness, *N* = 145 (72 lithic; 73 mineral); sphericity, *N* = 381 (171 lithic; 210 mineral); size, *N* = 373 (165 lithic; 208 mineral); colour, *N* = 131 (lithic).

Microscopy (optical and scanning electron) and Powder X-ray Diffraction (PXRD) were used to characterize the minerals present in the clast (lithic and mineral) and matrix materials, respectively. As required, energy dispersive spectroscopic data were collected using an Oxford Energy Dispersive Spectroscopy system, attached to FEI Apreo FEG-SEM. All compositional data were collected with beam parameters set to 20 keV, spot size 12. PXRD data were collected using a Bruker D8 discover-MR A25 equipped with Dectris Eiger2 R 500 K detector. The instrument uses a copper, Incoatec Microfocus Source (IμS) operating at 50 kV and 1 mA and is calibrated using a statistical approach^43^. The samples were pulverized and mounted as a 250 μm spherical powder ball with sample exposure time during data collection of 300 s over a range of 70°2θ. A total of 17 samples of matrix material, taken from various locations through the hand sample. Representative diffractograms are shown in Supplementary Fig. S4. In the initial stages of the investigation, compositional data were also collected by the electron microprobe operating in wavelength dispersive mode. Upon careful inspection and consideration, these data were found to not be germane to the discussion and are thus not presented here.

### Ontogenetic age determination

We were unable to sample our skeletons for skeletochronology, which is the preferred method of developmental age determination in fossil vertebrates^44^. Nevertheless, *P. lujiatunensis* is otherwise well-sampled in this regard, and Erickson et al.^17^ developed a simple linear equation for estimating the age of individuals of this species from femur length (age in years = 0.0615 * femur length [in mm]—1.9214; R^2^ = 0.9659). This yields an age of ~ 6.5 years for the *P. lujiatunensis* individual described here. Myhrvold^45^ subsequently showed that the growth analysis of Erickson et al.^17^ was flawed, but in their response, Erickson et al.^46^ did not update the above equation for deriving age from femur length. In their osteohistological analysis of *P. lujiatunensis*, Zhao et al.^18^ showed that IVPP V18343—among the largest individuals in their dataset (femur length = 132 mm [ref.^21^])—was an estimated 9 years of age. Given that the *P. lujiatunensis* individual we describe has a slightly longer femur, it stands to reason that it was correspondingly older, perhaps as old as 10 years. The obstructed teeth of the mammal meant that commonly used dental indicators for age estimation^19^ were not applicable.

### Body mass estimation

*Psittacosaurus lujiatunensis* was, in all probability, bipedal, at least in post-nestling individuals^47–49^. We therefore used the femur circumference-body mass scaling equation developed for bipedal non-avian vertebrates by Campione et al.^15^ and implemented in the ‘bipeds’ function in the R package MASSTIMATE^50^. However, because the preserved individual was not somatically mature, we used developmental mass extrapolation (DME)^16^ to volumetrically scale the mass of a fully mature individual down to that of a smaller one (assuming isometry of proportions). We used the femur circumference (reported as 69.362 mm) of a large *P. lujiatunensis* individual (Dalian Museum of Natural History specimen D2591) to estimate its mass at 23.5 ± 6.0 kg.

Extant-scaling approaches are used commonly in mammal palaeontology, so many different formulae have been derived for different skeletal/dental elements and taxa^51^. Given the relative immaturity of our *R. robustus* specimen, we estimated its body mass using DME as above, extrapolated from four commonly used estimation equations (Supplementary Table S1). (m1 area is a commonly used proxy for body mass, but measurements for the relevant *R. robustus* material were unavailable to us.) Of these four equations, combined stylopodial circumference exhibits the strongest relationship with body mass (lowest standard error of the estimate and percent prediction error, highest coefficient of determination), and so we accept the corresponding mass estimate in this study.

### Scaling of predator–prey size

We investigated the relationship between predator size and maximum prey size for both solitary and pack-hunting carnivorans using phylogenetic generalized least squares (PGLS) regression in R v. 4.0.2^52^ to provide some context for the fossil association described in this study. Body mass data for 78 carnivoran species were taken from the literature (Supplementary Information 2) and log-transformed to linearize correlations. Species relationships and branch lengths were taken from the maximum clade credibility tree of Slater and Friscia^53^, which was pruned as necessary using the drop.tip function in the ape package^54^. We generated several PGLS models assuming the following phylogenetic correlation structures: Brownian motion, Ornstein–Uhlenbeck (OU), Pagel’s λ, and Blomberg’s accelerated/decelerated (ACDC) transformation (assuming g = 0.5). These were modeled using the corClasses function in the ape package. Significant outliers were detected using Rosner’s test^55^, as implemented in the EnvStats package^56^, and deleted (yielding n_solitary_ = 64; n_pack_ = 12). We used the Akaike Information Criterion (AIC) to compare the fit of the models to our data^57^. Ninety-five percent confidence and prediction intervals were generated using the Evomap package^58^. Other methodological details as given in the original R script (Supplementary Information 2).

### Species diversity and abundance in the Lujiatun member

Zhang^5^ provided relative abundance data for various tetrapods from the Lujiatun Member, the information having been gathered by the Paleontological Experts Committee of Liaoning Province between 2001 and 2019. He reported that the total number of individuals in his tally was 1522, which we used to retrocalculate the original raw species counts (Supplementary Table S3). Because Zhang’s taxonomic survey of the Lujiatun Member was not exhaustive, we supplemented the species counts where necessary with additions from the literature, adding data for cf. *Euhelopus*, *Changmiania liaoningensis*, *Daliansaurus liaoningensis*, *Sinusonasus magnodens*, *Hexing qingyi*, *Incisivosaurus gauthieri*, *Liaoningvenator curriei*, *Shenzhousaurus orientalis*, Carnosauria indet., *Acristatherium yanensis*, *Gobiconodon zofiae*, *Juchilestes liaoningensis*, *Anebodon luoi*, *Meemannodon lujiatunensis*, and *Origolestes lii*. These data were subjected to rarefaction to assess sampling completeness in this member. The rarefaction curve was generated using the ‘individual rarefaction’ function in PAST v. 4.06b^59^, and the confidence interval was generated using an unconditional rarefaction variance estimate^60^.

Species abundance in vertebrate death assemblages rarely reflects that of the original biocoenosis because of the various taphonomic and collector biases that influence the detection and collection of bones; smaller skeletons tend to be destroyed preferentially by carnivore activity, bioturbation, and weathering, and are typically more difficult to detect on the landscape^61^. For this reason, some palaeoecological studies (e.g. ref.^38,62^) have attempted to correct for these biases with reference to a relationship describing the ratio of standing crop abundance on the landscape to skeletal abundance as a function of body size. The relevant data were collected for the mammal fauna from the semi-arid Amboseli Basin in southern Kenya^61^. The ratio of animals counted on the landscape (N) to skeletons observed (S) was expressed as a function of body mass in kg (W) as follows:

$${\text{log}}_{{{1}0}} \left( {{\text{N}}/{\text{S}}} \right) \, = { 1}.{96 }{-} \, 0.{\text{45 log}}_{{{1}0}} {\text{W }}\left( {{\text{R}}^{{2}} = \, 0.{82}} \right).$$

(The above equation was corrected by Coe et al.^62^ to exclude data from rhinoceros on account of their elevated death rates due to poaching.)

Importantly, the tetrapod fossil assemblage of the Lujiatun Member is unlike that of the Amboseli assemblage (and many fossil assemblages) in that it is not attritional; rather, it is preserved within an obrution deposit, which is not subject to the same external processes such as weathering, trampling, or carnivore activity^63^. Nevertheless, all fossil deposits, no matter their origin, are subject to observer/collector bias of the same sort that would influence the relationship established by Behrensmeyer et al.^61^ For this reason, we similarly opted to ‘correct’ our relative abundance data (Supplementary Table S3). Importantly, although the above equation relates to skeletons observed on the landscape, our fossil data include *Euhelopus* sp., which is represented only by isolated teeth in the Lujiatun Member^64^. It is therefore possible that the abundance/biomass of *Euhelopus* sp. is slightly overestimated here, but we felt it important to include because large-bodied animals have a disproportionate influence on the landscape^65^. We subsequently multiplied the corrected relative abundance data (expressed as a percentage of the overall tetrapod fauna) by the average per capita body mass of each species within the Lujiatun Member to derive an estimate of relative biomass, following Coe et al.^62^ These numbers were subsequently arranged by trophic level to reconstruct a biomass pyramid for the terrestrial vertebrates of the Lujiatun Member. See also Matsukawa et al.^66^ for a biomass pyramid of the entire Yixian Formation, which is much less temporally constrained than our own, but nevertheless has a similar shape.

## Supplementary Information

Supplementary Information 1.

Supplementary Information 2.

## Data Availability

The authors declare that the data supporting the findings of this study are available within the paper and its supplementary information files.
