# Author Correction: An extraordinary fossil captures the struggle for existence during the Mesozoic

**DOI:** 10.1038/s41598-024-72601-x

**Published:** 2024-09-12

**Authors:** Gang Han, Jordan C. Mallon, Aaron J. Lussier, Xiao‑Chun Wu, Robert Mitchell, Ling‑Ji Li

**Affiliations:** 1 Hainan Vocational University of Science and Technology, Haikou, Hainan China; 2 Hainan Tropical Ocean University, Sanya, Hainan China; 3 Beaty Centre for Species Discovery and Palaeobiology Section, Canadian Museum of Nature, Ottawa, Ontario Canada; 4 Department of Earth Sciences, Carleton University, Ottawa, Ontario Canada; 5 Beaty Centre for Species Discovery and Mineralogy Section, Canadian Museum of Nature, Ottawa, Ontario Canada; 6 Department of Geography, University of Calgary, Calgary, Alberta Canada; 7 Weihai Ziguang Shi Yan School, Weihai, Shandong China

Correction to: *Scientific Reports* 10.1038/s41598-023-37545-8, published online 18 July 2023

The original version of this Article contained an error in the legend of Figure 1.

“**Figure 1.** *Psittacosaurus lujiatunensis*-*Repenomamus robustus* pair (WZSSM VF000011) locked in mortal combat. Insets depict (left to right): hand of *R. robustus* wrapped around lower jaw of *P. lujiatunensis*, teeth of *R. robustus* embedded in forearm of *P. lujiatunensis*, hind foot of *R. robustus* wrapped around lower hindlimb of *P. lujiatunensis*. Scale bar equals 10 cm.”

now reads,

“**Figure 1.** *Psittacosaurus lujiatunensis*-*Repenomamus robustus* pair (WZSSM VF000011) locked in mortal combat. Insets depict (left to right): hand of *R. robustus* wrapped around lower jaw of *P. lujiatunensis*, teeth of *R. robustus* embedded in ribs of *P. lujiatunensis*, hind foot of *R. robustus* wrapped around lower hindlimb of *P. lujiatunensis*. Scale bar equals 10 cm.”

The original Article has been corrected.

