# Supplementary material for: An extraordinary fossil captures the struggle for existence during the Mesozoic
Source: Sci Rep. 2023 Jul 18;13:11221. doi: 10.1038/s41598-023-37545-8 (PMC10354204; doi:10.1038/s41598-023-37545-8)
Supplement: Supplementary file 1 — Supplementary Information 1. [file 41598_2023_37545_MOESM1_ESM.pdf]

An extraordinary fossil captures the struggle for existence during the Mesozoic

Gang Han[1,2], Jordan C. Mallon[3,4]*, Aaron J. Lussier[5], Xiao-Chun Wu[3], Robert Mitchell[6], Ling-Ji Li[7]

[1]Hainan Vocational University of Science and Technology, Haikou, Hainan, China.

[2]Hainan Tropical Ocean University, Sanya, Hainan, China.

[3]Beaty Centre for Species Discovery and Palaeobiology Section, Canadian Museum of Nature, Ottawa, Ontario, Canada.

[4]Department of Earth Sciences, Carleton University, Ottawa, Ontario, Canada.

[5]Beaty Centre for Species Discovery and Mineralogy Section, Canadian Museum of Nature, Ottawa, Ontario, Canada.

[6]Department of Geography, University of Calgary, Calgary, Alberta, Canada.

[7]Weihai Ziguang Shi Yan School, Weihai, Shandong, China.

*Corresponding author. Email: jmallon@nature.ca

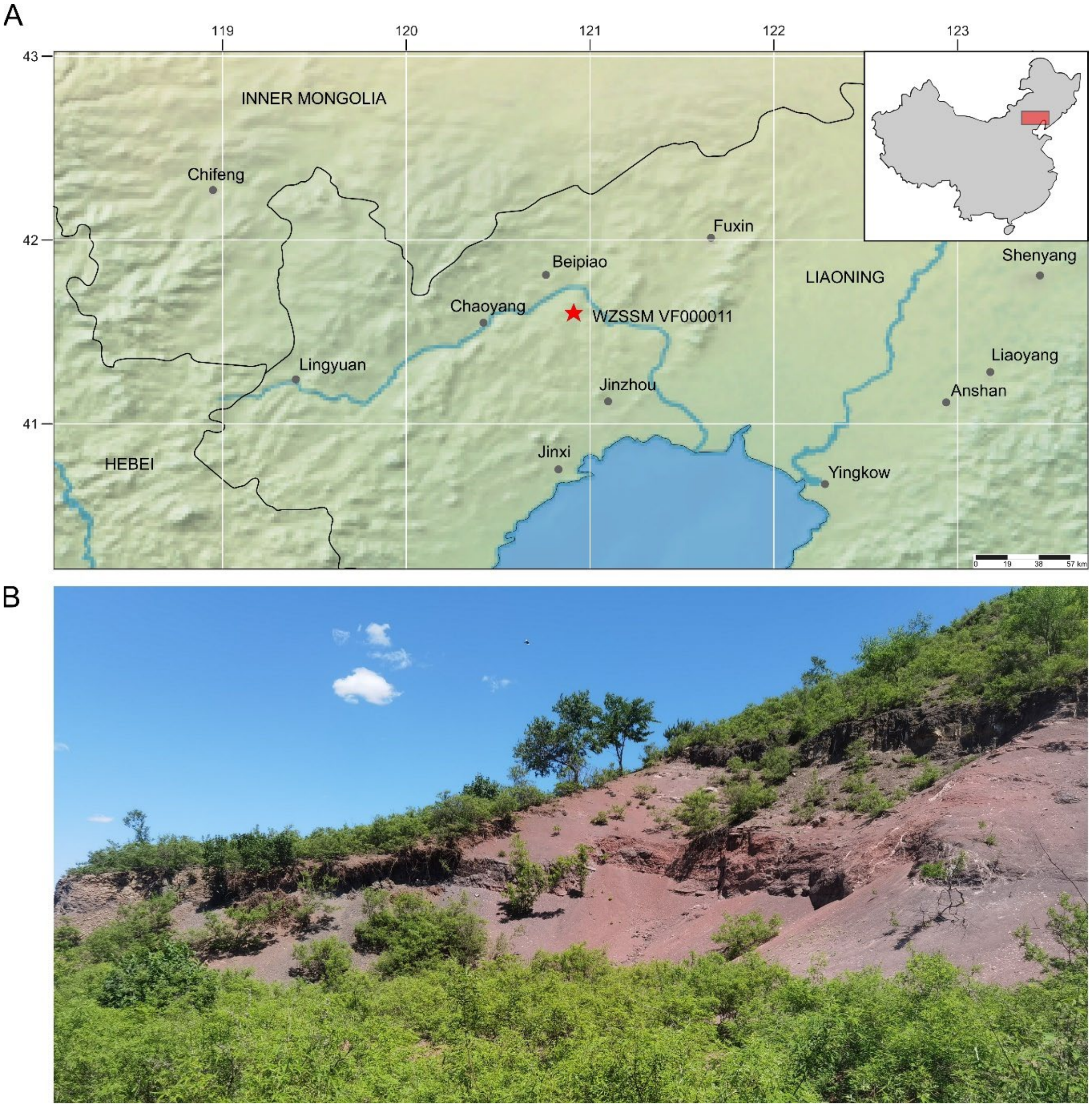

Figure S1. Locality details for WZSSM VF000011. (**A**) Map showing location of WZSSM VF000011 discovery site (red star) in northeastern China. Inset shows enlarged portion of mainland China. (**B**) Hillside where WZSSM VF000011 was collected from the Lujiatun Member of the Yixian Formation in 2012.

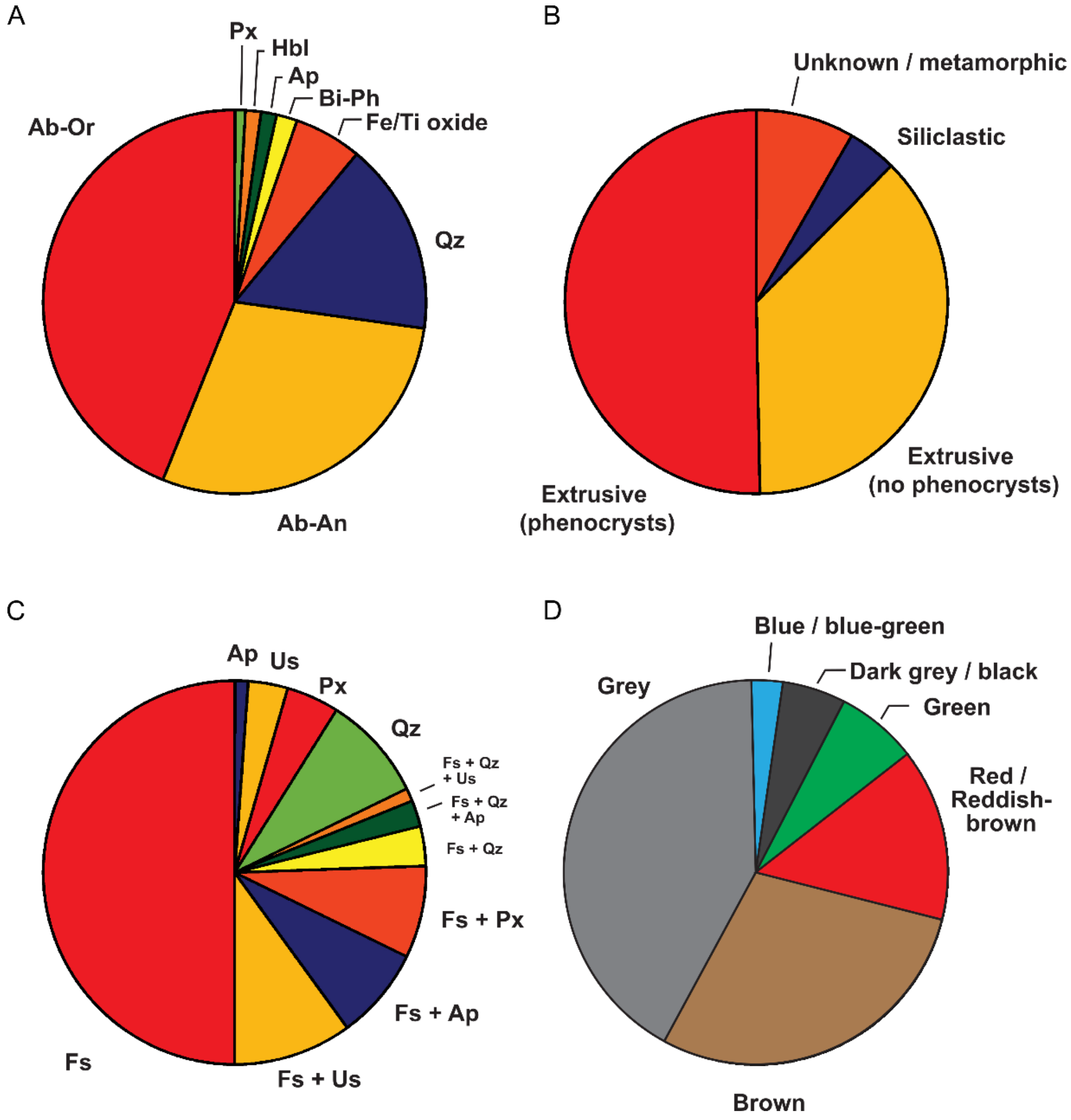

Figure S2. Pie charts showing the breakdown of investigated framework fragments from L-TS-2. (**A**) The distribution of mineral species in mineral fragments ($N = 228$). (**B**) Lithoclasts ($N = 169$) grouped by geologic origin. (**C**) The commonly observed grouping of phenocryst species among lithoclasts of extrusive origin ($N = 90$). (**D**) Lithoclasts grouped by predominant colour hue ($N = 131$). Note: colour groups are highly generalized to constrain complexity of pie chart, e.g., 'green' encompasses green-blue + green-yellow + dark green + light green, etc. Abbreviations: Fs, feldspar (An-Or-Ab); Us, ulvöspinel; Ap, apatite; Px, pyroxene; Qz, quartz; Hbl, hornblende; Bi, biotite; Ph, phlogopite.

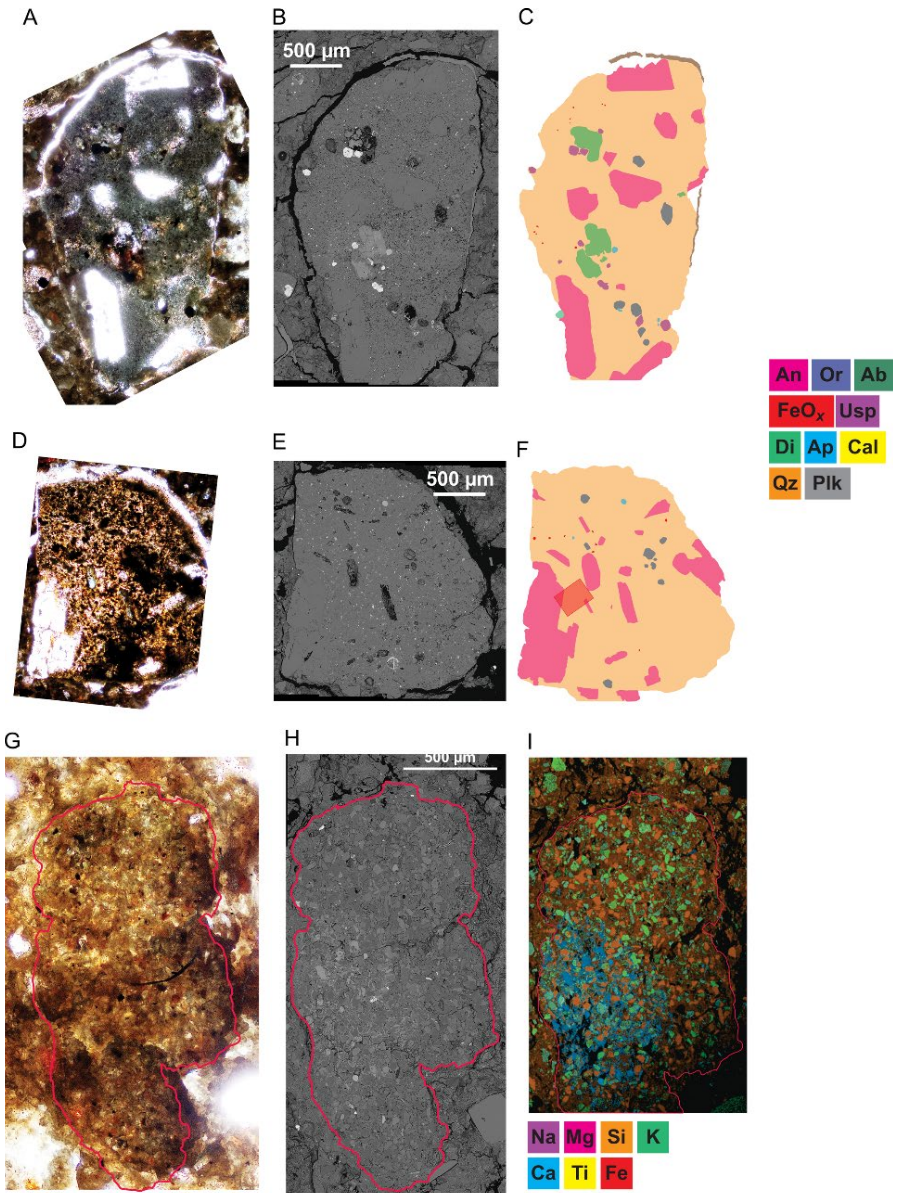

Figure S3. Images of selected lithoclast framework grains from thin section L-TS-2. (**A–C**) c63. (**D–F**) c180. (**G–I**) c157. For c63 and c180 (from left to right), optical image taken in plane-polarized light, backscatter electron micrograph, schematic illustration identifying location of major phases. For c157, optical image, electron micrograph, and energy dispersive X-ray map. Coloured boxes containing either mineral phases or chemical elements indicate phases (in **C** and **F**) and EDS regions (in I), respectively. Abbreviations: anorthite, An; orthoclase, Or; albite, Ab; iron oxides, FeOx; ulvospinel, Usp; diopside, Di; apatite, Ap; calcite, Cal; quartz, Qz, plucked / absent region, Plk. Lithoclasts c63 and c180 both consist of highly altered extrusive igneous material. The occurrence of multiple species of phenocrysts is observed in c63 (plagioclase, diopside-hedenbergite, and apatite). Conversely, in c180 only plagioclase phenocrysts are observed. In both clasts, individual phenocrysts are fragmented at the margins. Lithoclast c157 is a fragment of sedimentary material (lithic arenite), showing localized overgrowth of apatite of selected grains as well as intergranular apatitic cement (blue; Supplementary Fig. S4I).

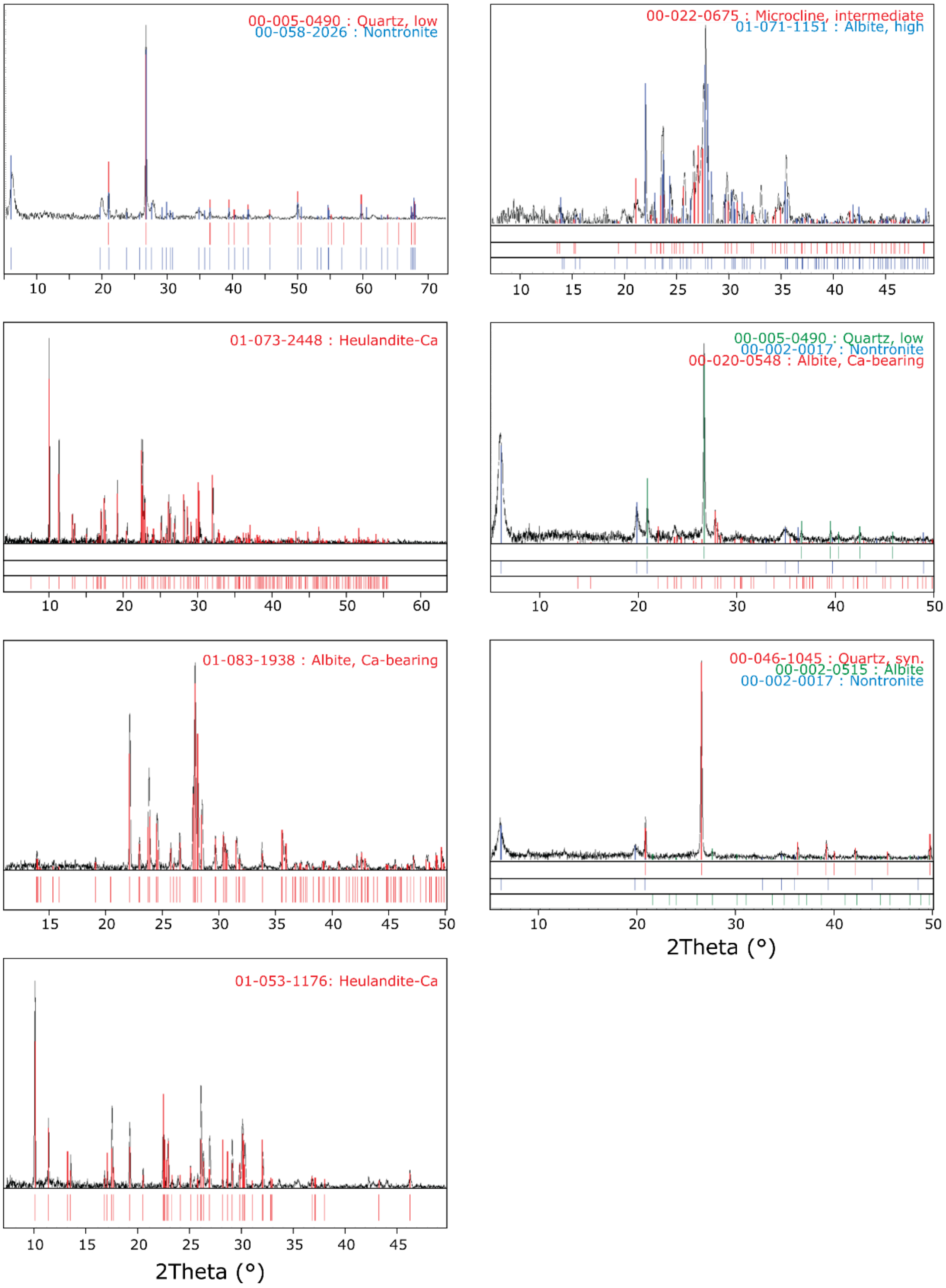

Figure S4. Selected powder X-ray diffractograms collected in matrix material from original hand sample. For each pattern, peak-matching results are shown at bottom.

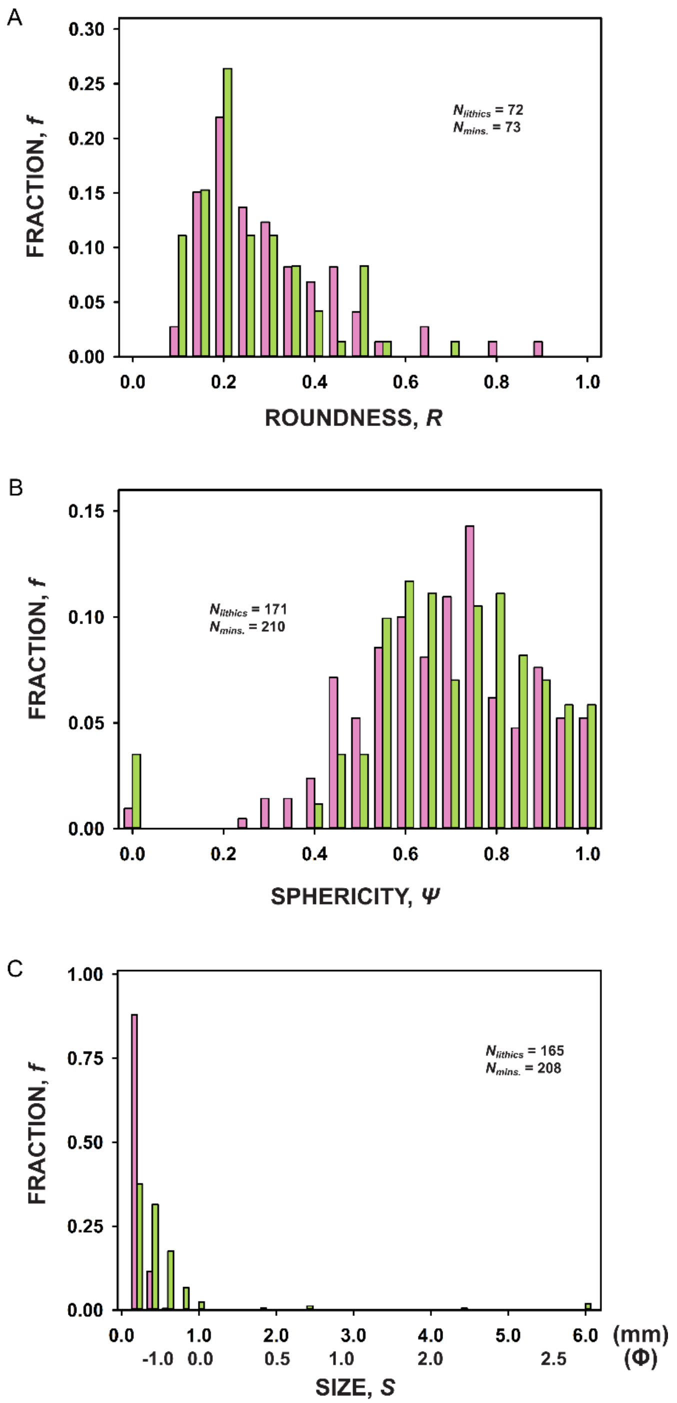

Figure S5. Histograms showing distributions of roundness, *R,* (**A**), sphericity, *ψ,* (**B**), and size, *S*, of constituent framework particles (**C**) in section L-TS-2. The distributions corresponding to mineral and lithic fragments are differentiated by colour. Framework fragments are highly angular and show a considerable range of roundness values ($0.1 < R \leq 0.9$). Observed sphericity values fall over the maximal possible range ($0 < \psi \leq 1$, for both lithic and mineral fragments). For mineral fragments, the observed size range is narrow (0.2–0.4 mm). By contrast, for lithic fragments, the observed size range is relatively broad; specifically, 96% of the clasts range between 0.2 and 1.0 mm, and clasts with larger dimensions (> 4.0 mm) are also observed.

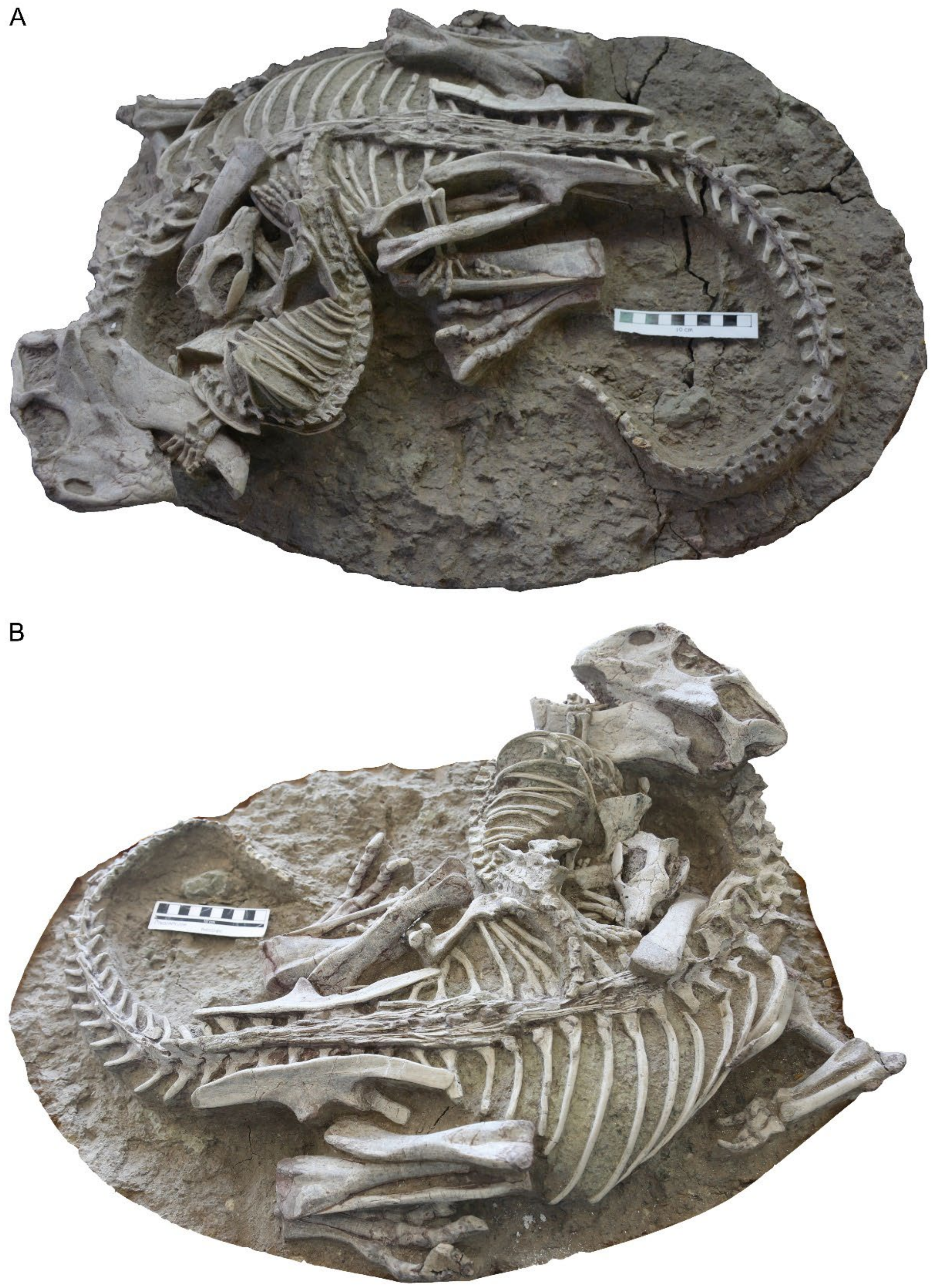

Figure S6. *Psittacosaurus lujiatunensis* and *Repenomamus robustus* individuals (WZSSM VF000011) locked in mortal combat. (**A**) Left side of fossil association. (**B**) Right side of fossil association. Scale bar equals 10 cm.

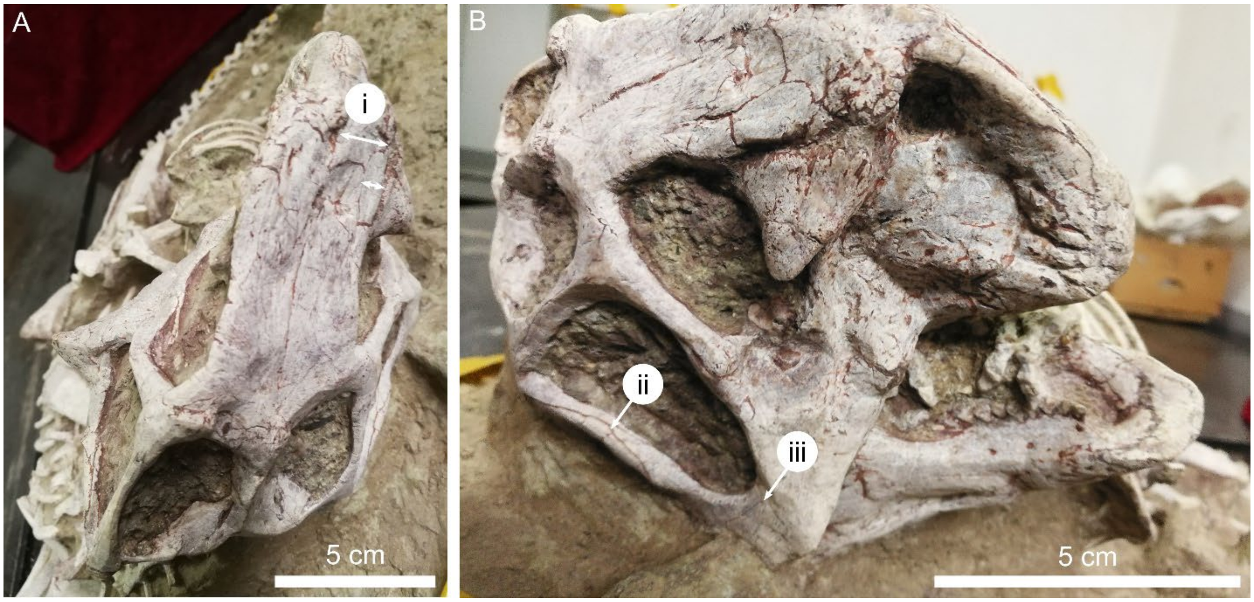

Figure S7. Taxonomically informative characters for *Psittacosaurus lujiatunensis* (WZSSM-VF000011). (**A**) Prefrontal width less than 50% that of the nasal (i). (**B**) Quadratojugal-squamosal contact along the anterior margin of the quadrate shaft (ii), and jugal-quadrate contact posteroventral to the laterotemporal fenestra (iii).

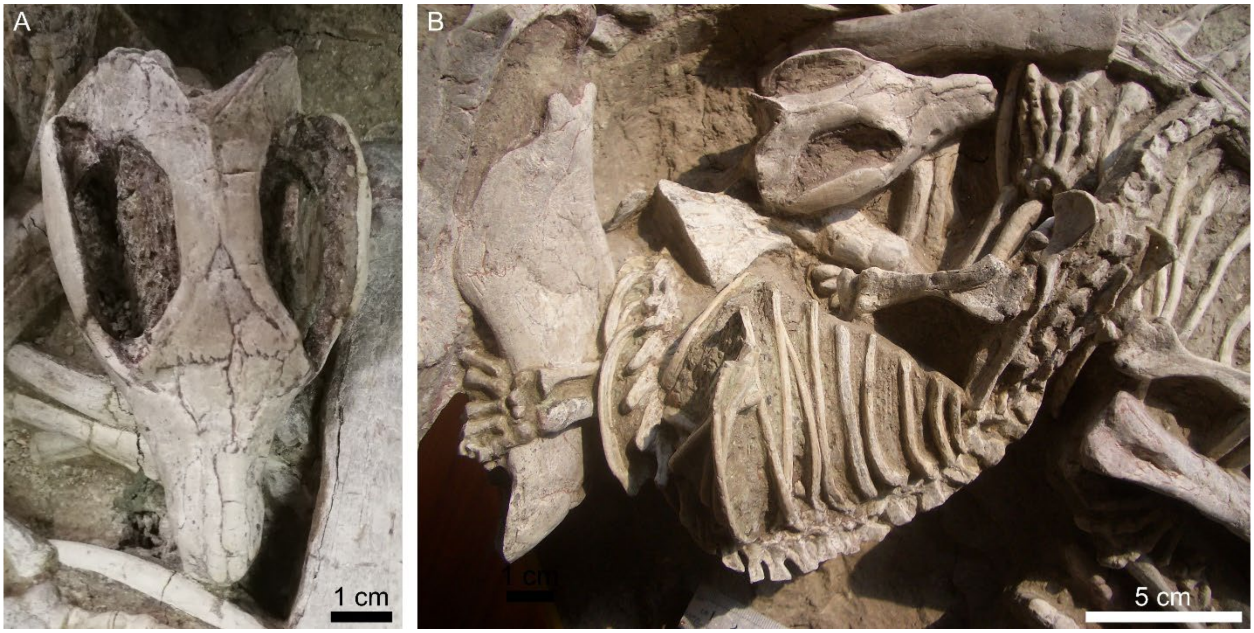

Figure S8. *Repenomamus robustus* (WZSSM-VF000011). (**A**) Dorsal view of skull. (**B**) Detail of postcranium.

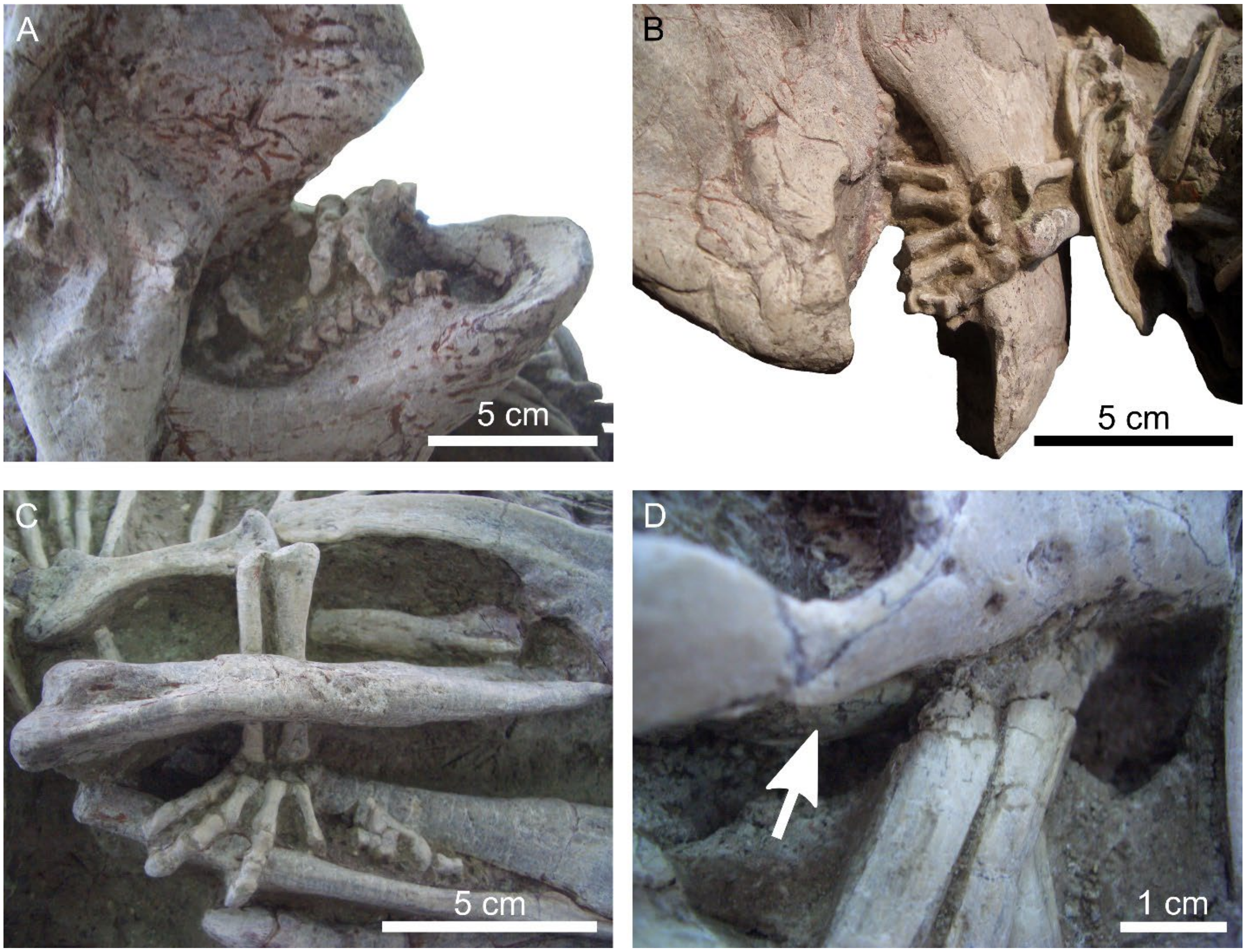

Figure S9. Details of *Psittacosaurus lujiatunensis-Repenomamus robustus* pair (WZSSM-VF000011). (**A**) Left hand of *R. robustus* clutching the lower jaw of *P. lujiatunensis* (right side). (**B**) Left hand of *R. robustus* clutching the lower jaw of *P. lujiatunensis* (left side). (**C**) Left hind foot of *R. robustus* grasping the left hindlimb of *P. lujiatunensis*. (**D**) Mandible of *R. robustus* plunging downward into the matrix to grip the dinosaur's ribs.

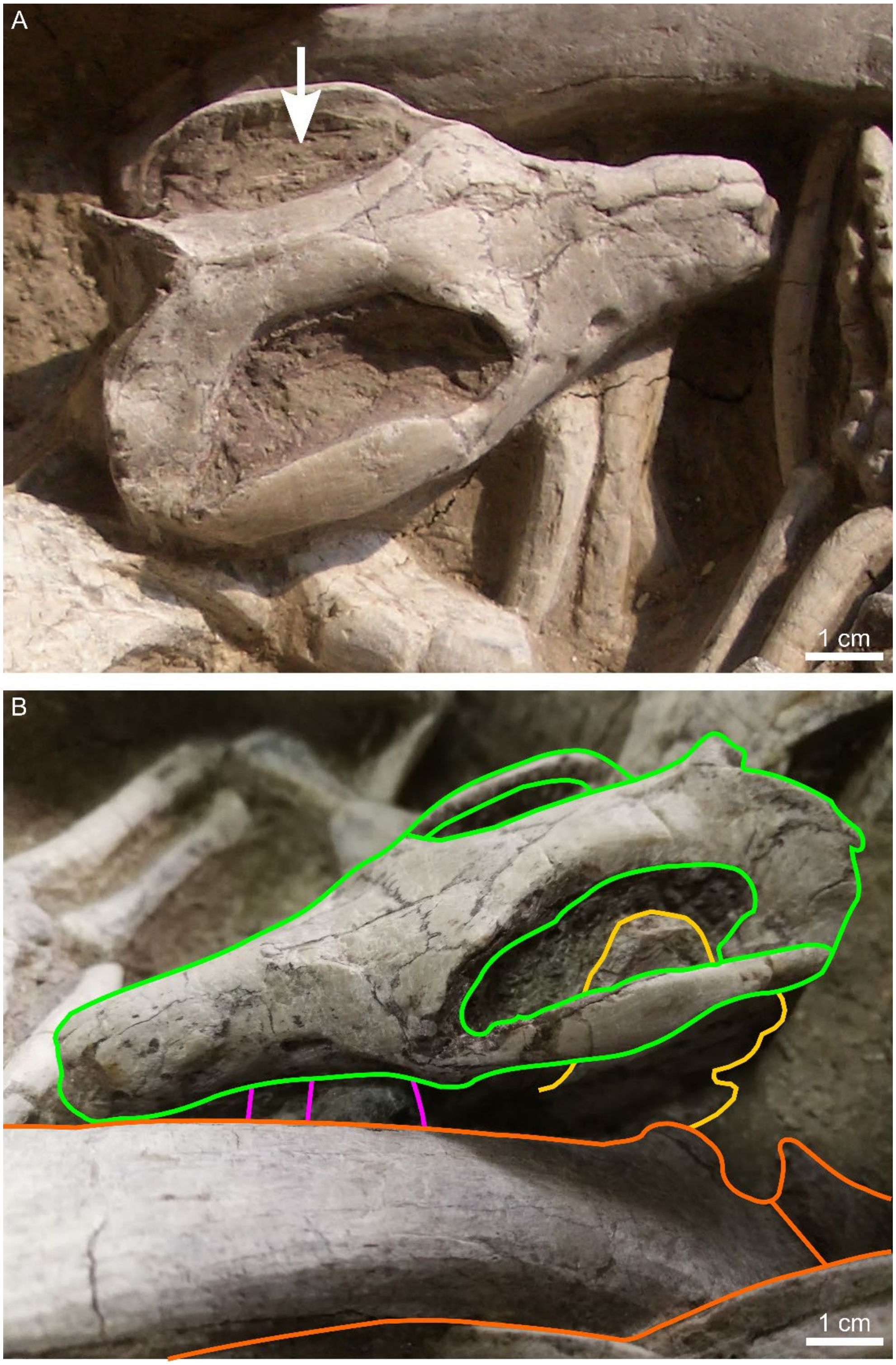

Figure S10. Preparation of the *Repenomamus robustus* individual (WZSSM VF000011). (**A**) Detail of the skull prior to additional preparation by WZSSM, showing the unexposed left temporal fossa (white arrow). (**B**) More recent image showing preparation of the temporal fossa and in situ left dentary. Coloured outlines: gold = left dentary of *R. robustus*, green = cranium of *R.robustus*, orange = left scapulocoracoid of *P. lujiatunensis*, purple = dorsal ribs of *P. lujiatunensis*.

Table S1. Commonly used extant-scaling formulae used to estimate body mass in mammals. Abbreviations: DME, developmental mass extrapolation; PPE, percent prediction error; SEE, standard error of the estimate.

Element	Source	Formula	R^2	SEE	PPE	Mass estimate (kg) for largest known specimen (catalogue # in brackets)	DME mass estimate (kg), scaled against femur length
dentary length (marsupials)	(*67*)	ln(body mass) = 2.9677 * ln(dentary length) - 5.6712	0.962	not reported	not reported	1.42 (IVPP V12549)	0.878
stylopodial circumference (quadrupedal terrestrial tetrapods)	(*68*)	$\log_{10}$(body mass) = 2.749 * $\log_{10}$(combined stylopodial circumference) - 1.104	0.988	0.134	25.6	5.54 (IVPP V12728)	3.43
skull length (carnivorans)	(*69*)	$\log_{10}$(body mass) = 3.13 * $\log_{10}$(skull length) - 5.21	0.903	0.220	47	6.67 (IVPP V12728)	4.13
head-body length (carnivorans)	(*69*)	$\log_{10}$(body mass) = 2.88 * $\log_{10}$(head-body length) - 7.24	0.922	0.185	36	2.08 (IVPP V13605)	1.41

Table S2. PGLS model parameters and performance. Abbreviations: AIC, Akaike Information Criterion.

**Model**	**Strategy**	**Parameter estimate**	**Slope (std. error)**	**t (slope)**	***p* (slope)**	**Intercept (std. error)**	**t (intercept)**	***p*(intercept)**	**Correlation (Intr)**	**AIC**
Brownian motion	Solitary	--	1.2300719 (0.0902849)	13.624341	0.000	-0.8228118 (0.9073170)	-0.906863	0.368	-0.365	158.076
	Pack	--	1.926414 (0.4179211)	4.609517	0.0010	-3.183547 (2.4657334)	-1.291116	0.2257	-0.716	46.03859
Ornstein-Uhlenbeck	Solitary	α = 15.95809	1.0077396 (0.1126727)	8.943958	0.0000	0.2144569 (0.4201652)	0.510411	0.6116	-0.982	129.4175
	Pack	α = 6.495951	2.307729 (0.3512437)	6.570164	0.0001	-4.709349 (1.4643124)	-3.216082	0.0092	-0.991	30.74509
Pagel' λ	Solitary	λ = 0.7531995	1.1181081 (0.1043521)	10.714764	0.0000	-0.3788819 (0.5124311)	-0.739381	0.4625	-0.741	126.5609
	Pack	λ = -0.6691558	2.042376 (0.427122)	4.781716	0.0007	-3.346608 (1.832070)	-1.826681	0.0977	-1	25.79092
ACDC	Solitary	g = 0.5 (assumed)	1.2262408 (0.0914294)	13.411887	0.0000	-0.7928084 (0.5819410)	-1.362352	0.178	-0.573	150.9835
	Pack	g = 0.5 (assumed)	1.944097 (0.4160424)	4.672834	0.0009	-3.253137 (2.0804547)	-1.563666	0.1490	-0.849	43.51615

Table S3. Raw and corrected relative abundance/biomass data used in this study. Abbreviations: BM, body mass; MNI, minimum number of individuals; N/S, number of individuals on landscape/number of skeletons.

Form	Taxon	Trophic level	Avg BM	Relative abundance (%) after (*51*)	Raw MNI (source)	Observed MNI (%)	Corrected MNI * Avg BM	Biomass (%)	N/S	N/S * Obs MNI	Corrected MNI (%)	Corrected MNI * Avg BM	Corrected biomass (%)
sauropod	cf. *Euhelopus*	Primary consumer	5924142		10 (*66*)	0.643915	3814644	62.91856	0.081718	0.05262	0.027176	160991.8	12.95552
ornithischian	*Changmiania liaoningensis*	Primary consumer	3470		2 (*67*)	0.128783	446.877	0.007371	2.327297	0.299716	0.154789	537.1195	0.043224
ornithischian	*Jeholosaurus shangyuanensis*	Primary consumer	9663.981	0.0138	21.0036	1.352453	13070.08	0.215577	1.467844	1.98519	1.025258	9908.075	0.797334
ornithischian	*Liaoceratops yanzigouensis*	Primary consumer	2000	0.0012	1.8264	0.117605	235.2093	0.00388	2.982198	0.35072	0.181131	362.2614	0.029152
ornithischian	*Psittacosaurus lujiatunensis*	Primary consumer	23500	0.8954	1362.799	87.75266	2062187	34.01363	0.984055	86.35346	44.59754	1048042	84.33926
theropod	*Daliansaurus liaoningensis*	Secondary consumer	3401.81		1 (*68*)	0.064392	219.0476	0.003613	2.348175	0.151203	0.078089	265.6441	0.021377
theropod	*Graciliraptor lujiatunensis*	Secondary consumer	1756.553	0.0007	1.0654	0.068603	120.5043	0.001988	3.161568	0.216892	0.112015	196.7597	0.015834
theropod	*Mei long*	Secondary consumer	846.0785	0.0012	1.8264	0.117605	99.50275	0.001641	4.392029	0.516523	0.26676	225.7	0.018163
theropod	*Sinovenator changii*	Secondary consumer	1909.03	0.0062	9.4364	0.607624	1159.973	0.019133	3.045329	1.850415	0.955653	1824.371	0.146813
theropod	*Sinusonasus magnodens*	Secondary consumer	4780.045		1 (*69*)	0.064392	307.7942	0.005077	2.01491	0.129743	0.067006	320.2929	0.025775
theropod	*Hexing qingyi*	Secondary consumer	4173.941		1 (*70*)	0.064392	268.7663	0.004433	2.141678	0.137906	0.071222	297.2762	0.023923
theropod	*Incisivosaurus gauthieri*	Secondary consumer	2000		1 (*71*)	0.064392	128.783	0.002124	2.982198	0.192028	0.099174	198.3473	0.015962
theropod	*Liaoningvenator curriei*	Secondary consumer	2004.613		2 (*72*)	0.128783	258.1601	0.004258	2.979108	0.383658	0.198142	397.1976	0.031964
theropod	*Shenzhousaurus orientalis*	Secondary consumer	17267.65		1 (*73*)	0.064392	1111.89	0.018339	1.130433	0.07279	0.037593	649.1395	0.052238
theropod	*Dilong paradoxus*	Tertiary consumer	16820.47	0.0007	1.0654	0.068603	1153.93	0.019033	1.14386	0.078472	0.040527	681.6846	0.054857
theropod	undescribed carnosaur	Tertiary consumer	2541814		1 (*74*)	0.064392	163671.2	2.699585	0.119587	0.0077	0.003977	10108.55	0.813467
mammal	*Acristatherium yanensis*	Secondary consumer	26		1 (*75*)	0.064392	1.674179	2.76E-05	21.05048	1.355472	0.700038	18.20099	0.001465

Table S3 (continued).

Form	Taxon	Trophic level	Avg BM	Relative abundance (%) after (*51*)	Raw MNI (source)	Observed MNI (%)	Corrected MNI * Avg BM	Biomass (%)	N/S	N/S * Obs MNI	Corrected MNI (%)	Corrected MNI * Avg BM	Corrected biomass (%)
mammal	*Gobiconodon zofiae*	Secondary consumer	152		1 (*76*)	0.064392	9.787508	0.000161	9.509778	0.612349	0.31625	48.06995	0.003868
mammal	*Juchilestes liaoningensis*	Secondary consumer	101		1 (*77*)	0.064392	6.503542	0.000107	11.43024	0.73601	0.380115	38.39162	0.003089
mammal	*Anebodon luoi*	Secondary consumer	129.4118		1 (*78*)	0.064392	8.33302	0.000137	10.22379	0.658325	0.339994	43.99926	0.003541
mammal	*Maotherium asiaticus*	Secondary consumer	103.5	0.0007	1.0654	0.068603	7.10038	0.000117	11.30516	0.775565	0.400543	41.45621	0.003336
mammal	*Meemannodon lujiatunensis*	Secondary consumer	1959		1 (*79*)	0.064392	126.1429	0.002081	3.010125	0.193826	0.100102	196.1005	0.015781
mammal	*Repenomamus spp.*	Tertiary consumer	4521	0.0066	10.0452	0.646825	2924.298	0.048233	2.066067	1.336385	0.690181	3120.307	0.251101
mammal	*Origolestes lii*	Secondary consumer	58.95038		6 (*80*)	0.386349	22.77542	0.000376	14.56401	5.626791	2.905976	171.3084	0.013786
amphibian	*Liaobatrachus zhaoi*	Secondary consumer	36.6341	0.0078	11.8716	0.76443	28.00421	0.000462	18.04062	13.7908	7.122304	260.9192	0.020997
lizard	*Dalinghosaurus longidigitus*	Secondary consumer	94.695	0.0657	99.9954	6.438854	609.7273	0.010057	11.76665	75.76374	39.12844	3705.268	0.298175
