# Supplementary material for: An extraordinary fossil captures the struggle for existence during the Mesozoic
Source: Sci Rep. 2023 Jul 18;13:11221. doi: 10.1038/s41598-023-37545-8 (PMC10354204; doi:10.1038/s41598-023-37545-8)
Supplement: Supplementary file 2 — Supplementary Information 2. [file 41598_2023_37545_MOESM2_ESM.zip › Supplementary Information 2/Contents of Supplementary Information 2.docx]

| **File name** | **Description** |
| --- | --- |
| PGLS_raw_data.xlsx | Microsoft Excel Worksheet containing raw data used in PGLS analyses. |
| Mammal_max_data_pared_log.csv | Microsoft Excel Comma Separated Values file containing pared dataset used in PGLS analyses. |
| Carnivora_MCC.tre | TRE file describing carnivoran phylogeny used in the PGLS analyses. |
| PGLS_analysis.R | R file containing code used in PGLS analyses. |
